# Comparative genome analysis of Korean field strains of infectious laryngotracheitis virus

**DOI:** 10.1371/journal.pone.0211158

**Published:** 2019-02-07

**Authors:** Tae-Min La, Eun-Jung Choi, Joong-Bok Lee, Seung-Yong Park, Chang-Seon Song, In-Soo Choi, Sang-Won Lee

**Affiliations:** College of Veterinary Medicine, Konkuk University, Seoul, Republic of Korea; Oklahoma State University, UNITED STATES

## Abstract

Attenuated live infectious laryngotracheitis (ILT) virus (ILTV) vaccines have been used to prevent and control the outbreak of ILT worldwide. Recent studies using high-throughput sequencing technology have increased the number of complete genome sequences of ILTVs, enabling comparative genome analysis. Although 37 complete genome sequences of ILTV, including vaccine strains, have been reported, the complete genome sequence of any field strain of ILTV in South Korea is yet to be published. In this study, we determined and analyzed the complete genome sequences of three virulent Korean field strains of ILTV (40798/10/Ko, 0206/14/Ko, and 30678/14/Ko). Two of the Korean field strains (40798/10/Ko and 0206/14/Ko) displayed fewer non-synonymous single nucleotide polymorphisms than those of the Serva vaccine strain, indicating that these Korean field strains of ILTV most likely originated from the vaccine strain. The third ILTV strain, 307678/14/Ko, had two regions in the genome showing recombination between the Serva vaccine-like strain and the Australian A20 vaccine-like strain. Comparative genome analysis of ILTV using the Korean field strains with variable virulence can shed light on the recent trend of the emergence of virulent ILTV strains in the field. A few amino acid changes in the genome of ILTV vaccines could enhance the virulence in the vaccine strain, and natural recombination should be considered one of the major risks for the generation of revertant strains of ILTV under field conditions.

## Introduction

The double-stranded DNA virus family *Herpesviridae* is classified into three subfamilies—*Alphaherpesvirinae*, *Betaherpesvirinae*, and *Gammaherpesvirinae* [1]. Infectious laryngotracheitis virus (ILTV) is an alphaherpesvirus that is responsible for respiratory diseases in chickens worldwide. The clinical signs of ILTV infection include conjunctivitis, coughing, nasal discharge, expectoration of bloody mucus, and a reduction in egg production in layers [2].

The nucleotide sequence of a large genomic region of ILTV was first reported in 1990 [3]. Subsequently, nucleotide sequences of the remaining genomic regions were determined in multiple studies using different strains of ILTV [4–13]. The first complete genome sequence of ILTV was assembled by overlapping genome fragments of different virus strains [14]. After the introduction of high-throughput sequencing technology, the complete genome sequences of the attenuated ILTV vaccine and virulent strains were rapidly updated, and comparative genome analyses have been reported [15–23].

The ILTV genome, ranging from 150 to 155 kbp in length, is organized into four distinct regions: the unique long (UL) region, unique short (US) region, internal repeat (IR) region, and terminal repeat (TR) region. The viral genome encodes 79 predicted proteins [18,21]. Since 1982, ILTV has caused sporadic outbreaks in the poultry industry in Korea [24]. To help control the outbreaks, chicken embryo origin (CEO) live attenuated vaccines have been extensively used in breeder and layer flocks. CEO vaccines are well known to be able to convert to virulent strains through bird-to-bird passages [25], and virulent field strains genetically related to CEO vaccines have been detected in Korea [26–28]. Molecular epidemiology has played an important role in controlling infectious disease. However, progress in molecular epidemiology for ILTV has been slow in Korea owing to a lack of genetic information on the Korean field strains of ILTV.

In this study, we isolated three field strains of ILTV in Korea and determined the complete genome sequences of these strains. We conducted comparative genome and recombination analyses of the obtained genomic information of the Korean field strains.

## Materials and methods

### Animal ethics

Animal experiments were conducted in strict accordance with the guidelines of the animal and plant quarantine agency and institutional animal care and use committee. The experiment protocol was approved by the Institutional Animal Care and Use Committee of Konkuk University [IACUC approval number: KU16151].

### Virus isolation

Three Korean field strains of ILTV were isolated from diagnostically tested homogenized chicken tracheal samples from broiler and layer flocks showing respiratory symptoms and up to 30% mortality. The homogenized samples diagnosed as ILTV positive were inoculated onto chorioallantoic membranes (CAMs) of 10-day-old embryonated specific pathogen-free eggs, and the CAMs showing pocks were harvested 5 days after inoculation. Each viral strain was purified by plaque picking and subculturing three times in LMH cells (a chicken hepatoma cell line), followed by the propagation of the purified virus with additional passages on CAMs. The Korean field strains of ILTV were named 0798/04/Ko, 0206/14/Ko, and 30673/14/Ko.

### Purification of viral DNA for high-throughput sequencing

Each viral strain was passaged again in LMH cells for DNA extraction. The LMH cells showing cytopathic effects were frozen and thawed. The virus-infected LMH cells were briefly centrifuged, and the cell pellet was sonicated five times with 15 s pulses. The sonicated cells were centrifuged at 1,000 ×*g* for 15 min. The supernatant was pelleted by ultracentrifugation at 17,000 rpm for 2 h using a Beckman 19 Ti rotor (Beckman Instruments, Palo Alto, CA, USA). The pellet was dissolved in 3 ml of phosphate-buffered saline (PBS) and then dialyzed using a tube with a 1,000 kDa pore size (Spectrum). A beaker was filled with 100 ml of PBS, and the tube was gently stirred with a magnetic stirrer. The PBS was replaced with fresh PBS after 2 h and 16 h. The dialyzed virus was transferred to 1.5 ml microcentrifuge tubes. Viral DNA was extracted with an automatic nucleic acid extractor (SeePrep12 Instrument CE/IVD, Cat. SPN1200) and SeePrep12 Viral NA Kit (Cat. SPN1004) based on the manufacturer’s protocol.

### High-throughput sequencing and genome assembly

High-throughput sequencing was performed using an ion-torrent sequencing platform with a 318 chip at the Bio-core Lab (Seoul, South Korea) following the manufacturer’s instructions. Raw reads obtained from the Korean field strains of ILTV were mapped to the complete genome sequence of the Serva strain of ILTV (GenBank accession number HQ630064) using the Geneious mapper with default parameters, within Geneious software (ver. 7.0). Predicted coding regions and locations of genomic features were annotated with Geneious software (ver.7.0) [29]. Multiple genome alignment between the Korean field strains and 16 selected complete genome sequences of ILTV obtained from GenBank, listed in Table 1, was performed using the MAFFT (ver.7.388) method [30]. The maximum likelihood phylogenetic tree for the complete genome alignment was obtained with the T92+G+I model, selected as the best substitution model, and 500 bootstrap replicates using MEGA7 [31]. Recombination analysis was performed using the SimPlot software (ver.3.5.1) and SplitsTree (ver. 4.14.6). The terminal repeat region was deleted for the recombination analysis.

**Table 1:** Details of the complete genome sequences of ILTVs in GenBank used in this study.

Strains	Origin	GenBank accession number	Total length (bp)
LT Blen	USA	JQ083493	153494
Laryngo-Vac	USA	JQ083494	153495
Serva	Australia	HQ630064	152630
A20	Australia	JN596963	152978
V1-99	Australia	JX646898	153630
LJS09	China	JX458822	153201
WG	China	JX458823	153505
K317	China	JX458824	153639
Nobilis Laryngo-Vac	Italy	KP677881	153,650
193435/07	Italy	KP677883	153662
757/11	Italy	KP677884	153662
4787/80	Italy	KP677885	153653
O	Russia	KU128407	153624
40798/10/Ko	Korea	MH937566	153649
0206/14/Ko	Korea	MH937564	153645
30678/14/Ko	Korea	MH937565	153659

### Examination of pock size from CAMs inoculated with ILTVs

Sizes of pocks produced by inoculation of the three Korean field strains and the commercial vaccines Serva and LT Blen were compared. The inoculated CAMs were harvested 5 days post-inoculation, and the sizes and number of pocks on the CAMs were measured. Size differences of pocks were analyzed by one-way analysis of variance [32]. The data reflect the results of a Newman–Keuls multiple comparisons post-hoc test.

## Results

### Complete genome sequences of three Korean field strains of ILTV

Complete genomes of three Korean field strains of ILTV were determined using high-throughput sequencing. A total of 2,12,357, 5,21,787, and 98,6975 raw reads were obtained for 40798/10/Ko, 0206/14/Ko, and 30678/14/Ko, respectively. Among them, 22,741, 22,014, and 71,072 reads were mapped to the reference genome sequence with mean coverage rates of 28, 20, and 70 for 40798/10/Ko, 0206/14/Ko, and 30678/14/Ko, respectively (Table 2). After mapping analysis, homopolymeric errors were curated manually, and any ambiguous genomic regions were confirmed using PCR and Sanger sequencing. We observed that two Korean field strains, namely 0206/14/Ko and 40798/10/Ko, had genome sizes of 1,53,645 bp and 1,53,649 bp, respectively, while the 30678/14/Ko strain had a slightly longer genome, of 1,53,659 bp (Table 2). All genomes were observed to have 48.1% GC content and encode 78 coding sequences (CDSs).

**Table 2:** Results of the high-throughput sequencing run and genomic assembly.

Strains	Total number of reads	Number of mapped reads	Mean of coverage	Complete genome length (bp)	Unique long region (bp)	Unique short region (bp)	Internal and terminal repeat regions (bp)
40798/10/Ko	2,12,357	22,741	28	1,53,649	1,12,915	13,094	13,820
0206/14/Ko	5,21,787	22,014	20	1,53,645	1,12,915	13,094	13,818
30678/14/Ko	9,86,975	71,072	70	1,53,659	1,12,914	13,097	13,824

### Comparative analysis of ILTV strains

**Fig 1:**
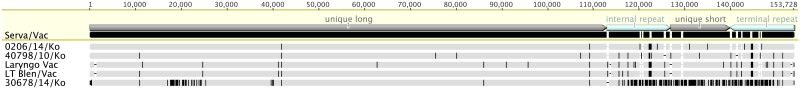
Complete genome alignment between the Korean field strains of ILTV and ILTV vaccine strain. The Serva vaccine was used as a reference. The alignment was performed using MAFFT. Vertical black lines represent single-nucleotide differences, and dashes represent gaps.

In multiple genome alignment between the Korean field strains and commercially available vaccine strains of ILTV, the 40798/10/Ko and 0206/14/Ko strains were genetically closer to the Serva vaccine than to the Laryngo-Vac or LT Blen vaccine strains (Fig 1). In total, 18 and 23 single nucleotide polymorphisms (SNPs) were detected between the Serva vaccine and the two Korean field strains (0206/14/Ko and 40798/10/Ko), respectively, by complete genome alignment. A major fraction of the complete genome of the 30678/14/Ko strain also showed close genetic relationship to the Serva vaccine. However, clustered SNPs were found in two large genomic regions of the 30678/14/Ko strain (Fig 1).

**Fig 2:**
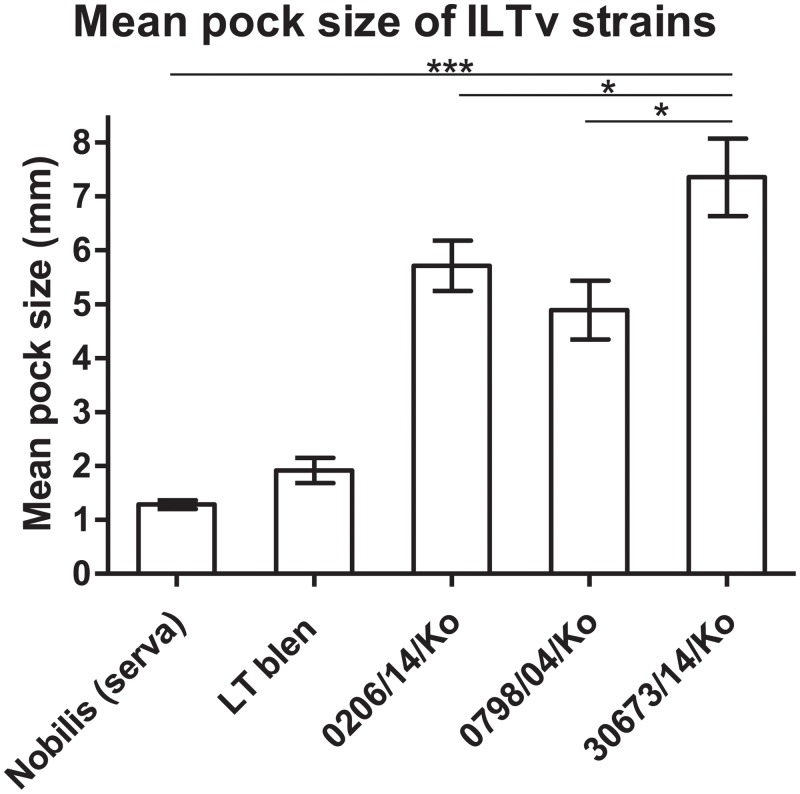
Comparison of the size of pocks produced by the Korean field strains and vaccines of ILTV on the CAM. The mean pock size on CAMs induced by each strain was measured 5 days post inoculation. Significant differences (P-values: * = P < 0.05, ** = P < 0.01, and *** = P < 0.001) were determined by the Newman–Keuls multiple comparisons post-hoc test.

The ability of the cell-to-cell spread of ILTV, which is considered one of the major characteristics related to the virulence of ILTV, has been assessed by pock sizes on CAMs [15]. Higher passaged viruses in tissue culture produced smaller pocks on the CAM compared with lower passaged viruses [33]. The pathogenicity of the Korean field strains was evaluated using CAM inoculation using CEO vaccine strains as control. Among all tested strains, the 30678/14/Ko strain produced the largest pocks, with a mean pock size of 7.4 mm. Although their genomic sequences were almost identical to that of the Serva vaccine strain, the 40798/10/Ko strain (4.88 mm) and the 0206/14/Ko strain (5.52 mm) also produced significantly larger pocks compared with those produced by the Serva strain (1.3 mm) or the LT Blen strain (1.98 mm) (Fig 2).

**Fig 3:**
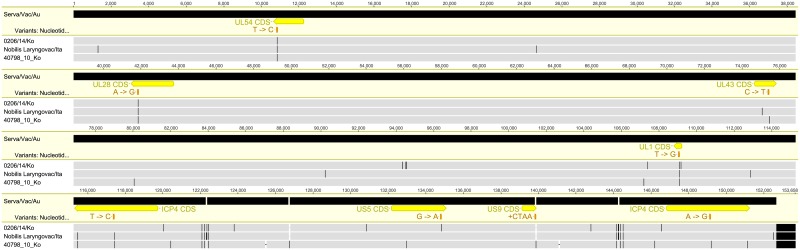
Complete genome alignment between 0206/14/Ko, 40798/10/Ko, and two attenuated Serva vaccine strains. The Serva vaccine was used as a reference. The alignment was performed using MAFFT. Locations of non-synonymous SNPs in the CDSs between the two Korean strains and vaccine strains are indicated. Vertical black lines represent single-nucleotide differences and dashes represent gaps.

Two complete genome sequences of the attenuated Serva vaccine strains have been determined until now [16,23]. We performed complete genome alignment between 40798/10/Ko, 0206/14/Ko, and two attenuated Serva strains to detect a genomic region associated with pathogenic reversion of the Serva vaccine strain (Fig 3).

Compared with the genome of the Serva vaccine strain, 18 and 23 SNPs were detected in the genomes of the 0206/14/Ko and 40798/10/Ko strains, respectively. Interestingly, 28 SNPs, including four non-synonymous SNPs, were detected between two ILTV vaccine strains containing the same attenuated strain but sold in different countries. Non-synonymous SNPs were confirmed using PCR and Sanger sequencing. Non-synonymous SNPs detected in the genome alignment are listed in Table 3. When compared with the genome of the Serva vaccine strain, the 0206/14/Ko strain showed a unique single-amino acid change, which mapped partially in the *US5* gene, and the 40798/10/Ko strain had a unique single-amino acid change in the *UL43* gene and showed a frame shift in the *US9* gene as a result of a four-nucleotide addition (Fig 3 and Table 3).

**Table 3:** Non-synonymous SNPs against the Serva vaccine strain of ILTV.

		0206/14/Ko			40798/10/Ko			Nobilis Laryngo-Vac/Ita		
Genes	Products	CDS position	SNPs	Amino acid change	CDS position	SNPs	Amino acid change	CDS position	SNPs	Amino acid change
UL54	Multifunctional expression regulator	1,437	T →C Partial (37/63%)	I → M	1,437	T → C	I → M	1,437	T → C	I → M
UL28	DNA packaging terminase subunit 2	1,913	A → G	V → A	1,913	A → G	V → A	1,913	A → G	V → A
UL43	Envelope protein UL43				761	C → T	T → I			
UL1	Envelope glycoprotein L	161	T → G	Q → P	161	T → G	Q → P	161	T → G	Q → P
ICP4	Transcriptional regulator ICP4				2,342	T → C	H → R	2,342	T → C	H → R
US5	Envelope glycoprotein J	2,662	G → A Partial (45/55%)	A → T						
US9	Membrane protein US8A				716	+CTAA	Frame shift			
ICP4	Transcriptional regulator ICP4				2,342	A → G	H → R	2,342	A → G	H → R

### Evidence of recombination of the 30678/14/Ko strain

In contrast to the two Korean field strains having almost identical genomic sequences to that of the Serva vaccine strain, the 30678/14/Ko strain showed two clustered SNPs (Fig 1). Results from the Blastn analysis using the clustered SNP genomic regions suggested that the A20 Australian-origin ILT vaccine strain has the highest nucleotide sequence identities with the 30678/14/Ko strain.

**Fig 4:**
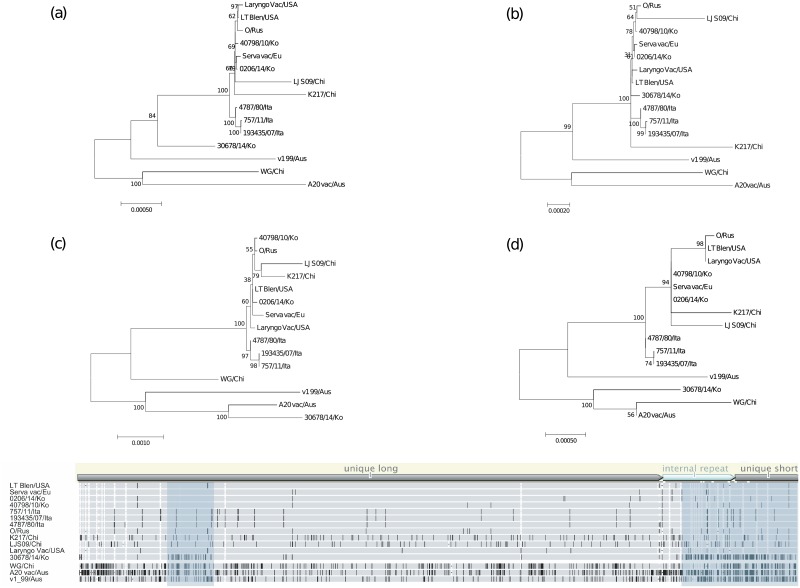
Phylogenetic tree analyses for the 30678/14/Ko strain of ILTV using alignments of the complete genome and sub-regions of the complete genome between ILTV strains. Multiple genome alignment was performed using MAFFT. Maximum likelihood phylogenetic trees using the T92+G+I model were generated using alignments of the complete genome (a) and three genomic regions showing clustered SNPs in the unique long (b) and the internal repeat and unique short regions (d), which are indicated as shaded boxes in genome alignment, and the middle of the genomic region (c). Five hundred bootstrap replications were used to assess the significance of the tree topology. Bars indicate nucleotide substitutions per site.

**Fig 5:**
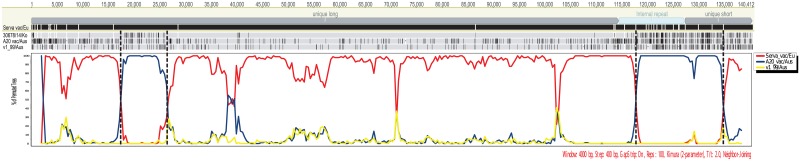
Recombination analysis for the 30678/14/Ko strain of ILTV. Bootscan analyses were performed using SimPlot with 30678/14/Ko as a query. The crossover points of predicting recombination sites are indicated on the alignment of the complete genome sequence without a terminal repeat region with a color-coded dashed line.

We performed a phylogenetic tree analysis to elucidate the relationship between the A20 and 30678/14/Ko strains. Four phylogenetic trees were generated using multiple nucleotide alignments, including the complete genome, two of the clustered SNP regions (in the unique long, internal repeat, and unique short regions), and the middle of the clustered SNP region, between the 15 strains of ILTV. In the tree using the complete genome, the 30678/14/Ko strain did not cluster with the Serva or A20 strain (Fig 4a). In the tree using the middle of the clustered SNP region, the 30678/14/Ko strain closely clustered with the Serva vaccine strains and other Korean field strains (Fig 4b). However, in two other trees using the clustered SNP regions, the 30678/14/Ko strain was found to be genetically close to the A20 strain (Fig 4c and 4d). To define recombination crossover points, Bootscan analysis was performed using complete genome alignment between four ILTV strains: Serva and A20 as potential parental sequences, the 30678/14/Ko as a query sequence, and V1-99 as a control. Consistent with results of the phylogenetic tree analysis, the Bootscan analysis showed two pairs of crossover points upstream and downstream from the clustered SNP regions (Fig 5). Pair-wise homoplasy test using SplitsTree detected recombination in complete genome alignment, the unique long region, and the internal repeat region, but not in the unique short region (Table 4).

**Table 4:** Results of recombination analysis by the pair-wise homoplasy index (PHI) test using SplitsTree.

	Informative site	Mean	*P-*value
Complete genome	194	0.529	0.0
Unique long region	125	0.418	0.0
Internal repeat region	50	0.312	1.035E-12
Unique short region	19	0.432	0.1003

## Discussion

Live attenuated CEO ILTV vaccines have been extensively used worldwide to prevent and control laryngotracheitis infections. However, the vaccine itself can convert to virulent strains through bird-to-bird passages and outbreaks [34]. Therefore, understanding the molecular epidemiology of ILTV is essential to distinguish the revertant field strains from attenuated CEO vaccine strains [35]. In the past, PCR and restriction fragment length polymorphism (RFLP) targeting multiple genomic regions have been used to classify ILTV strains. The increased availability of high-throughput sequencing technology at lower costs has enabled the determination of the complete genome sequence of ILTV and the elucidation of the genetic evolution and relationships among ILTV strains. Comparative genome analyses of closely related strains of ILTV or strains isolated from specific geographical regions have been previously reported [36–38].

In this study, complete genome sequences of three Korean field strains of ILTV were determined, and comparative genome analysis of these field strains and other reference strains was performed. Results from the complete genome analysis indicated that the 0206/14/Ko and 40789/10/Ko strains are genetically similar to the Serva vaccine strain. In a previous study, 24 SNPs were identified by complete genome alignment between the SA2 and A20 vaccine strains [15]. The A20 vaccine strain was highly attenuated by 20 sequential passages of the SA2 strain, having residual virulence in chicken embryonic kidney cell cultures [39]. The number of SNPs in the 0206/14/Ko and 40789/10/Ko strains compared with that in the Serva vaccine strain were only 18 and 23, respectively, which indicated that these Korean field strains have very likely originated from the Serva vaccine strain. These results suggested that the two Korean field strains of ILTV originated from the Serva vaccine and became revertant by nucleotide substitutions. The origin of several virulent field strains from vaccine strains have been reported previously [18]. However, the genetic alterations responsible for the reacquisition of virulence have not been studied so far. Only single-amino acid changes in different CDS regions were detected between these two Korean field strains and the Serva vaccine. Alanine changed to threonine in glycoprotein J encoded by the *US5* gene of the 0206/14/Ko strain, and threonine changed to isoleucine in the envelope protein UL43 encoded by the *UL43* gene of the 40798/10/Ko strain. Furthermore, a frame shift was detected in the membrane protein encoded by the *US9* gene. ILTV glycoprotein J (gJ) is a major viral antigen required for the efficient egression of the viral particles from infected cells [40]. The deletion of gJ has been implicated in *in vitro* growth defects and *in vivo* attenuation [35]. The ILTV *US9* gene encodes a predicted type II membrane protein, and its homologs in other alphaherpesviruses are responsible for spreading to neurons [41,42]. Unlike other alphaherpesviruses, the ILTV US9 protein is a minor protein. The deletion of the *US9* gene does not affect replication kinetics; it only moderately reduces plaque sizes in CEK cells [43]. The *UL43* gene is predicted to encode a transmembrane protein and is only present in alphaherpesviruses and gammaherpesviruses [44]. The *UL43* gene product showed membrane fusion inhibition of viral fusion machinery similar to gM in the pseudorabies virus [45]. These changes might be determinants of reversion to virulence for vaccine strains.

In a previous study, the MSD CEO vaccine and LJS09 strain showed the same amino acid change as that of the *UL43* gene of 40798/10/Ko. In addition, the SNP in the *UL43* gene in the Serva vaccine strain was detected in the mapped reads, indicating the presence of a subpopulation in the vaccine strain [23]. In this study, only 40798/10/Ko had the SNP found in the subpopulation of the Serva vaccine strain. This suggests that both the major and minor populations of strains from the vaccine can revert to the virulent strain.

Although all the Korean field strains may have been derived from commercial vaccines, the 30678/14/Ko strain produced larger pocks on CAMs than did the other two Korean field strains. Comparative genome analysis and recombination analysis revealed that 30678/14/Ko is a natural recombinant, having multiple recombined genomic regions between the Serva-like and A20-like strains. In an earlier study, a mosaic genomic pattern of 30678/14/Ko between global CEO vaccines and Australian-origin vaccines was detected using PCR and Sanger sequencing of multiple genomic regions [46]. Recently, recombination events between two attenuated ILTV vaccines resulting in more virulent or transmissible field strains have been reported [47]. Two Australian virulent field strains, created by independent recombination events between the Serva and A20 vaccines, had different recombined genomic regions when compared with the 30678/14/Ko strain. These results indicated that there is no specific genomic region that can increase the virulence of ILTV through recombination between the Serva-like and A20-like strains. In Korea, only the Serva vaccine has been used, but Australian vaccines have not been imported here before. Genomic regions genetically close to the Australian vaccine strains have also been detected in other recombinants isolated in the USA and China, although the Australian vaccines have not been exported overseas [48,49]. The frequent detection of multiple virulent recombinants of ILTV worldwide suggests that natural recombination might be a common evolutionary strategy employed by ILTVs to facilitate their survival in host populations.

Comparative genome analysis of ILTV using the Korean field strains, having variable virulence, can allow for better understanding of the recent emergence trend of virulent ILTV strains in the field. In the past, genetic changes in the genome of vaccine strains through bird-to-bird passages were considered a major cause for revertant generation. However, natural recombination should be considered one of the major risks for the generation of revertant strains of ILTV in the field condition. Further comparative genome analysis of the two Korean field strains, 0206/14/Ko and 40798/10/Ko, might provide clearer insights into the mechanisms by which genetic determinants allow vaccine strains to reacquire virulence.
